# The abundance of health-associated bacteria is altered in PAH polluted soils—Implications for health in urban areas?

**DOI:** 10.1371/journal.pone.0187852

**Published:** 2017-11-16

**Authors:** Anirudra Parajuli, Mira Grönroos, Sari Kauppi, Tomasz Płociniczak, Marja I. Roslund, Polina Galitskaya, Olli H. Laitinen, Heikki Hyöty, Ari Jumpponen, Rauni Strömmer, Martin Romantschuk, Nan Hui, Aki Sinkkonen

**Affiliations:** 1 Department of Environmental Sciences, Section of Environmental Ecology, University of Helsinki, Lahti, Finland; 2 Finnish Environment Institute, SYKE, Centre for Sustainable Consumption and Production, Contaminants, Helsinki, Finland; 3 Department of Microbiology, University of Silesia, Katowice, Poland; 4 Kazan Federal University, Kazan, Russia; 5 Department of Virology, School of Medicine, University of Tampere, Tampere, Finland; 6 Fimlab Laboratories, Pirkanmaa Hospital District, Tampere, Finland; 7 Division of Biology, Kansas State University, Manhattan, Kansas, United States of America; Universite Paris-Sud, FRANCE

## Abstract

Long-term exposure to polyaromatic hydrocarbons (PAHs) has been connected to chronic human health disorders. It is also well-known that i) PAH contamination alters soil bacterial communities, ii) human microbiome is associated with environmental microbiome, and iii) alteration in the abundance of members in several bacterial phyla is associated with adverse or beneficial human health effects. We hypothesized that soil pollution by PAHs altered soil bacterial communities that had known associations with human health. The rationale behind our study was to increase understanding and potentially facilitate reconsidering factors that lead to health disorders in areas characterized by PAH contamination. Large containers filled with either spruce forest soil, pine forest soil, peat, or glacial sand were left to incubate or contaminated with creosote. Biological degradation of PAHs was monitored using GC-MS, and the bacterial community composition was analyzed using 454 pyrosequencing. Proteobacteria had higher and Actinobacteria and Bacteroidetes had lower relative abundance in creosote contaminated soils than in non-contaminated soils. Earlier studies have demonstrated that an increase in the abundance of Proteobacteria and decreased abundance of the phyla Actinobacteria and Bacteroidetes are particularly associated with adverse health outcomes and immunological disorders. Therefore, we propose that pollution-induced shifts in natural soil bacterial community, like in PAH-polluted areas, can contribute to the prevalence of chronic diseases. We encourage studies that simultaneously address the classic “adverse toxin effect” paradigm and our novel “altered environmental microbiome” hypothesis.

## Introduction

Pollutants, such as polyaromatic hydrocarbons (PAHs), can lead to shifts in microbial communities [1–4]. PAH degradation typically lasts for decades in polluted environments including soil, water, air, and sediments [5–7]. Direct PAH toxicity, abiotic transformation, and microbial degradation favor some microbial taxa, whereas others become less prevalent in PAH-polluted soils [8]. The bacterial taxa that thrive under PAH exposure vary depending on the types of soil and environmental conditions [9–12]. A search for general trends in microbial shifts in PAH-polluted soils necessitates concurrent studies of microbial communities in different soil types.

Exposure to PAHs is associated with severe human health deficits and ecological impacts, such as process of PAH-DNA adduct formation and carcinogenesis [13], and therefore they have been classified as priority environmental pollutants by the United States Environmental Protection Agency (US-EPA) and the Environmental European Agency (EEA) [14, 15]. The environmental concentration of PAHs and the connection between direct PAH exposure and human health have been well investigated [16–23]. Almost 90% of the PAHs released into the atmosphere accumulate in surface soil layers [14] where they are primarily degraded by soil bacteria or bind to soil particles [22]. The health outcomes of PAH-exposure have been attributed to direct and indirect toxin effects on humans [23, 24].

Several studies have investigated the relationship between human health and the most common bacterial taxa found in the human microbiome. A rich or dominant Proteobacterial and diminished Bacteroidetes communities in the human gut can contribute to a risk of immune system disorders including chronic obstructive pulmonary disease and asthma [25], and such effects can be transgenerational. For example women whose infants developed IgE-associated eczema had lower diversity of Bacteroidetes in their gut during the pregnancy [26]. Alteration in the Proteobacterial abundance in various regions of human body is associated with several health disorders, but its effects are twofold. On the positive side, diverse Proteobacterial community on skin is related to a reduced risk of atopy [27, 28]. On the negative side, overgrowth of Proteobacteria is connected to asthma and chronic obstructive pulmonary disease [29]. Within Proteobacteria, Betaproteobacteria are enriched in the gut microbiome of individuals with type 2 diabetes [30]. Importantly, recent findings indicate that Proteobacterial community of individual’s skin depends on land use in their living environment and that the environment tunes response to allergens [27–31]. Soil is the major reservoir of Actinobacteria, Bacteroidetes, and Proteobacteria [32, 33], and thus the living environment likely tunes individual’s responses to allergens, sugar metabolism, and health disorders. For these reasons, it is possible that adverse health effects connected to living in contaminated environment may partly be attributable to fundamental changes in the environmental microbiome people are exposed to, in addition to the direct physiological responses caused by the toxins. Surprisingly, to the best of our knowledge, no studies have focused on the potential connection between PAH pollution and soil community changes in the abundance of health-associated bacteria.

In the present study, we sampled four different surface soils, contaminated them with PAHs and followed population shifts in bacterial community in contaminated and non-contaminated containers under controlled conditions. Finally, based on previous empirical evidence as well as our new findings and novel ideas, we propose a new “altered environmental microbiome” hypothesis that should be investigated in parallel with the traditional “direct toxin effect” paradigm as a potential explanation for the complex relationship between human health and environmental pollution.

## Materials and methods

### PAH source

We selected creosote as the contaminant because it is among the most widely used wood preservatives with a history of more than a century in wood impregnation industry [5]. Creosote consists of 85% PAHs which are chemical compounds consisting of at least two aromatic rings fused together [34, 35]. The remaining is less than 10% phenolic compounds and 5–10% heterocyclic aromatic compounds consisting of oxygen, sulphur, and nitrogen. Therefore, creosote is a relevant compound to test environmental changes caused by PAH-pollution since it consists mainly of PAHs and is a common problem in urban areas with a history of sawmills.

### Soil collection

Surface soil was collected at four separate sites in Finland. The first site (Peat in Table 1) was at Haapasuo peat production area in Leivonmäki, Finland [36] (61°54’N 26°4’E). The current surface layer was formed hundreds of years ago, and it represents the transition layer between minerotrophic *Carex* and ombrotrophic *Sphagnum* dominated peat bogs. The original bog was drained, and the water table has been kept 30–40 cm below the peat surface for more than three decades. The second site, referred to as pine forest soil, is a boreal pine (*Pinus sylvestris* L.) forest in Hollola, located in southern Finland (61°0’N 25°29’E) moraine ridge (the undisturbed ecosystem has been described before [36]). The site is characterized by a thin organic soil layer on top of mineral soil [37]. The third site (Spruce forest soil in Table 1) is a spruce forest in Vierumäki, southern Finland (60°52’7N 25°41’E). Spruce (*Picea abies*) is the dominant tree species, and the field layer consisted of *Sphagnum* and *Pleurozium schreberi* mosses as well as dwarf shrubs, mainly *Vaccinium* species. The fourth site (sand) was next to (distance 50 m) the site 3 (Spruce forest soil), but it consisted of bare glacial sand and scattered ruderal herbs as original surface soil was removed years earlier when the site became a storage field for lumber.

**Table 1:** The pH and organic matter content for each soil type.

	Peat	Pine forest	Spruce forest	Sand
**pH**	3.52 ± 0.03^b^	3.6 ± 0.26^b^	3.25 ± 0.04^c^	4.87 ± 0.05^a^
**Organic matter (%)**	97.75±0.41^a^	63.42 ± 8.15^b^	35.19 ± 12.41^c^	1.21 ± 0.17^d^

Values are mean ± 1 SD. Letters in the superscript denote statistical differences in Tukey’s tests among different soil types

At all four sites, soil was collected from three separate (distance >5m) ca. 1 m^2^ plots as described in earlier work [38, 39]. In short, at each plot, live vegetation and plant debris were removed and 15–20 L of surface soil (depth 2–15 cm) was collected and mixed thoroughly. The soil was then randomly divided into two 10 L polyethylene containers, and the procedure was repeated at each 1 m^2^ plot. Filled containers were covered with polyethylene lids with two 5 mm diameter holes sealed loosely with cotton wool to facilitate passive aeration. Soil weight in containers varied between 3–12 kg depending on soil type, the heaviest being the mineral soil-types.

#### Basic physicochemical analyses

Moisture content was measured by drying samples in an oven (+ 90°C) for 24 hours. Organic matter (OM) was determined as loss of ignition at 550°C for 4h. Nutrient contents of the different soil types were determined with QuikChem 8000 flow injection analysis system (LACHAT Instruments Inc., USA) [38]. To measure the pH, 10g (fresh weight) of each soil type was mixed in 50 mL of 1M CaCl_2_, shaken for 5 min and allowed to settle for 2–24 hours [38].

#### PAH degradation experiment

For each soil type, three pairs of 10L polyethene containers were included. In the beginning of the experiment, soil was mixed thoroughly within pairs in large polyethene containers and thereafter divided again into two 10L polyethene containers. Of each pair, one container was randomly chosen to be spiked with creosote, whereas another received 100g glacial sand (Lohja Rudus Oy, Lahti, Finland). Initial (day 0) samples for basic chemical analyses were acquired immediately after spiking. Creosote spiking was done as described earlier [38]. In short, creosote (6–24 g) was mixed thoroughly with 100g glacial sand, and the mixture was incorporated into soil in the containers selected for contamination. This resulted in the concentration of ~ 1% PAHs similar to those in creosote contaminated sites. After creosote addition, containers were left to incubate for four weeks (28 days) at 16 ± 1°C and sampled at days 28, 91, and 189 for chemical and bacterial community analyses. Each sample (total weight 10 g) consisted of five subsamples from four depths (2, 5, 10, and 15 cm) collected from each container. Community sequencing analyses were performed on day 0 and when the total PAH concentration had decreased by more than 20% from week four (day 28) value. Therefore, week 13 (day 91) samples were analyzed in most cases. However, week 27 (day 189) samples were utilized for each of control and contaminated mineral soil containers because their PAH concentrations at week 13 were more than 95% of week 4 concentrations. To avoid the transfer of microorganisms between containers, the sampling equipment was carefully flame sterilized with 70% ethanol right before taking each sample.

### PAH analyses

PAH concentrations were determined using toluene extraction as described in Nordic Guidelines for Chemical Analysis of Contaminated Soil Samples [40] and analyzed as described earlier [41] with the exception that aqueous sodium pyrophosphate decahydrate solution (0.05 M) instead of hexane was used. The extracts were analyzed with Shimadzu GC–MS-QP5000 system equipped with AOC-20i autoinjector and 30-m ZB-5MS column (0.25 mm i.d., 0.25 μm film thickness). The oven program was set as follows: 80°C for 1 min, 10°C/min to 250°C, 7°C/min to 280°C, 20°C/min to 320°C with a hold of 10 min for a total run time of 34.29 min. PAH-mix 9 (16 PAHs included) was used for GC-MS and PAH-Mix 31 of five deuterated PAHs for soil samples (Dr. Ehrenstorfer, GmbH Germany) as standards and Anthracene-D10 (Dr. Ehrenstorfer, GmbH Germany) as a recovery standard.

### DNA extraction, amplification, and sequencing

Total DNA was extracted from each soil sample using FastDNA SPIN Kit for Soil (MP Biomedicals, Illkirch, France) according to the manufacturer’s standard protocol. The highly hypervariable region V3 of bacterial 16S rRNA gene was amplified using primer constructs that incorporated the pyrosequencing adapters (A), sample-specific DNA tags, and MF341 5’ CTA CGG GAG GCA GCA G 3’ or R518 5′ ATT ACC GCG GCT GCT GG 3′ [42]. The PCR were conducted under the following conditions: 200 nM of each forward and reverse primers, 5 ng template DNA, 200 μM of each dNTP, 2.5 mM MgCl_2_, 1 U GoTaq Hot Start DNA polymerase (Promega, Madison, WI), and 2.5 μl PCR buffer. The PCR cycle parameters consisted of an initial denaturation at 94°C for 3 min, then 25 cycles of denaturation at 94°C for 1 min, annealing at 54°C for 1 min, and extension at 72°C for 2 min, followed by a final extension step at 72°C for 10 min. The PCR products were purified using Ampure XP Magnetic Clean-up (Agencourt Bioscience Corporation, Beverly, MA, USA), and quantified using Nanodrop (Thermo Scientific, Rockford, IL, USA) and Bioanalyzer 2100 with DNA 1000 chips (Agilent Technologies Inc., Santa Clara, CA, USA). The sequencing was performed using the 454 GS FLX protocol and the GS FLX Titanium Rapid Library Preparation Kit (454 Life Sciences, Roche Diagnostics, CT, USA). The bacterial sequence data are available in the Sequence Read Archive at NCBI under accession number SRR5229978.

#### Sequence analysis

The sequence data were analyzed using MOTHUR (v1.35.0, 64-bit for Linux) according to the standard operating protocol as described earlier [43]. Briefly, the raw sequence data were quality controlled, and reads with ambiguous bases or homopolymers longer than 8 bp (722 sequences) were removed. The UCHIME algorithm [44] identified 943 sequences as chimeric, and these were subsequently omitted. Sequences were assigned into Operational Taxonomic Units (OTU) at 97% similarity and OTUs assigned to taxon affinities using Naïve Bayesian Classifier [45] against the RDP training set (version 10). Rare OTUs occurring three or fewer times across all samples were omitted to avoid problems caused by uncertainty in origin.

#### Statistical analyses

The relative abundance was calculated as the number of sequences in a taxon divided by total number of sequences in a sample. To compare contaminated and pristine treatments, *t*-test on relative abundances of bacterial phyla was conducted. Significant results are indicated by asterisks (p<0.05). False Detection Rate (FDR) was used for the p-values correction in *t*-tests. The difference in the pH and the organic matter content across the four soil types was calculated using ANOVA in JMP (v.11.0 64-bit; SAS Institute, Cary, North Carolina). To visualize bacterial community compositions of whole bacterial communities as well as the major bacterial phyla, non-metric multidimensional scaling (NMDS) analyses was performed based on relative abundance of OTUs using vegan package in R (v3.2.2, R Development Core Team 2015). The Bray-Curtis distance was chosen in the NMDS analysis since there were null values between samples in the data [46]. We performed the NMDS analyses at 99, 97, 95, 93, 91% OTU similarity levels but only present the data at 97% because OTU threshold had no impact on the overall conclusions.

Differences in the bacterial community composition between creosote contaminated and control groups were tested using permutative analysis of variation (PERMANOVA, function *adonis* in R-package *vegan* and Bray-Curtis metric). The difference in the relative abundances of bacterial phyla between creosote contaminated and control soils was calculated using the T-test in JMP.

## Results

### Soil chemical characteristics

The four soil types used in this study differed in pH (F = 164.4, df = 3, 20, *p* < 0.001) and organic matter content (F = 183.2, df = 3, 20, *p* < 0.001). Sandy soil had the highest and spruce forest soil the lowest pH (Table 1). Organic matter content decreased in the following order: peat > pine forest soil > spruce forest soil > sandy soil (Table 1). Thus, the four soil types represent a range of habitats with highly variable bacterial communities.

### Characteristics of bacterial operational units (OTUs) and relative abundances of bacterial phyla

The pyrosequenced bacterial 16S rRNA gene fragment datasets were analysed from 24 soil samples taken on days 0, 91, and 189. Day 0 samples for microbiological analyses were taken before contamination. Then the time point at which 20% or more of PAH concentration on day 28 had disappeared was determined. In sand, 20% degradation was reached on day 189 and in other soils on day 91 (S1 Table). Day 28 was used as a reference day because the reduction in PAH concentration until day 28 is typically caused by the evaporation of naphthalene and other low molecular weight compounds [47, 48].

In total 3626 OTUs were obtained, which represented more than 20 bacterial phyla from different soil types with and without PAH contamination. Bacterial OTUs representing Proteobacteria were the most abundant group accounting for 50.9%, 31.5%, and 35.8% of the total sequences in contaminated, pristine, and day 0 samples, respectively (Table 2). Likewise, Proteobacteria was the predominant phylum when compared across the different soil types with 46.20% (peat soil), 37.7% (pine forest soil), 36.0% (sand), and 37.8% (spruce forest soil) of all sequences. Other dominant phyla in all soils were Acidobacteria, Actinobacteria, Verrucomicrobia, and Bacteroidetes (Tables 2 and 3).

**Table 2:** Relative abundances of bacteria phyla (classes) on day 0 and when 20% of PAH contamination had disappeared, i.e. days 91 and 189 in sand and other soils (both pristine and contaminated), respectively.

	Week 0		No creosote		Creosote added	
Phylum	Mean	1SD	Mean	1SD	Mean	1SD
Acidobacteria	0.240	0.110	0.269	0.089	0.268	0.170
Actinobacteria	0.150	0.060	0.092*	0.041	0.049*	0.016
Armatimonadetes	0.001	0.001	0.002	0.002	0.000	0.000
Bacteroidetes	0.048	0.026	0.069*	0.034	0.010*	0.013
Candidate_division_WPS-1	0.000	0.000	0.001	0.002	0.000	0.000
Candidatus_Saccharibacteria	0.014	0.010	0.013*	0.006	0.002*	0.003
Chlamydiae	0.001	0.001	0.004	0.007	0.001	0.002
Chloroflexi	0.002	0.003	0.002	0.004	0.001	0.001
Fibrobacteres	0.001	0.003	0.002	0.003	0.000	0.000
Firmicutes	0.008	0.010	0.015	0.015	0.031	0.062
Gemmatimonadetes	0.030	0.060	0.007	0.013	0.001	0.003
Nitrospirae	0.001	0.004	0.002	0.003	0.001	0.002
Parcubacteria	0.001	0.001	0.003*	0.003	0.001*	0.000
Planctomycetes	0.004	0.005	0.005	0.005	0.004	0.006
Proteobacteria	0.358	0.077	0.315*	0.071	0.509*	0.166
Alphaproteobacteria	0.100	0.026	0.120	0.033	0.127	0.075
Betaproteobacteria	0.165	0.035	0.082*	0.032	0.234*	0.057
Gammaproteobacteria	0.043	0.021	0.054	0.019	0.076	0.018
Deltaproteobacteria	0.047	0.015	0.057	0.041	0.061	0.022
Unclassified proteobacteria	0.004	0.002	0.003	0.001	0.010	0.003
Spirochaetes	0.003	0.008	0.009	0.022	0.000	0.001
Unclassified bacteria	0.081	0.070	0.130	0.107	0.049	0.061
Verrucomicrobia	0.040	0.030	0.060	0.035	0.074	0.054

To compare contaminated and pristine treatments, we conducted *t*-test between creosote added and no creosote.

Significant differences indicated by asterisk (p<0.05).

P-values corrected by FDR.

**Table 3:** The effects of soil type and creosote contamination on bacterial community composition at genus and OTU levels in PERMANOVA.

Genus level	Df	Sum of squares	Mean square	F Model	R^2^	P value
**Soil type (1)**	3	0.09	0.03	4.81	0.38	0.001
**Creosote addition (2)**	1	0.06	0.06	9.08	0.24	0.001
**1:2**	3	0.04	0.01	2.16	0.17	0.014
**Residuals**	8	0.05	0.01		0.21	
**Total**	15	0.24			1.00	
**OTU level**						
**Soil type (1)**	3	2.02	0.67	2.74	0.37	0.001
**Creosote addition (2)**	1	0.35	0.35	1.41	0.06	0.078
**1:2**	3	1.07	0.36	1.45	0.20	0.028
**Residuals**	8	1.98	0.25		0.36	
**Total**	15	5.41			1.00	

The relative abundances of different phyla between contaminated and pristine treatments were compared. Proteobacteria (df = 1, *p* = 0.046) were more abundant in contaminated soils, whereas the relative abundances of Actinobacteria (df = 1, *p* = 0.043) and Bacteroidetes (df = 1, *p* = 0.005) declined in contaminated soils (Table 2). In addition, it was observed that the relative abundances of Parcubacteria and Candidus Saccharibacteria were lower in the contaminated soils, although the relative abundances of these two were very low (Table 2). As taxa belonging to Proteobacteria represented more than 35% of microbial community in all soil types, we compared the relative abundances of the classes under the phylum Proteobacteria and found that the relative abundance of Betaproteobacteria was higher (df = 1, *p* = 0.013) in contaminated soils.

### Bacterial community composition

**Fig 1:**
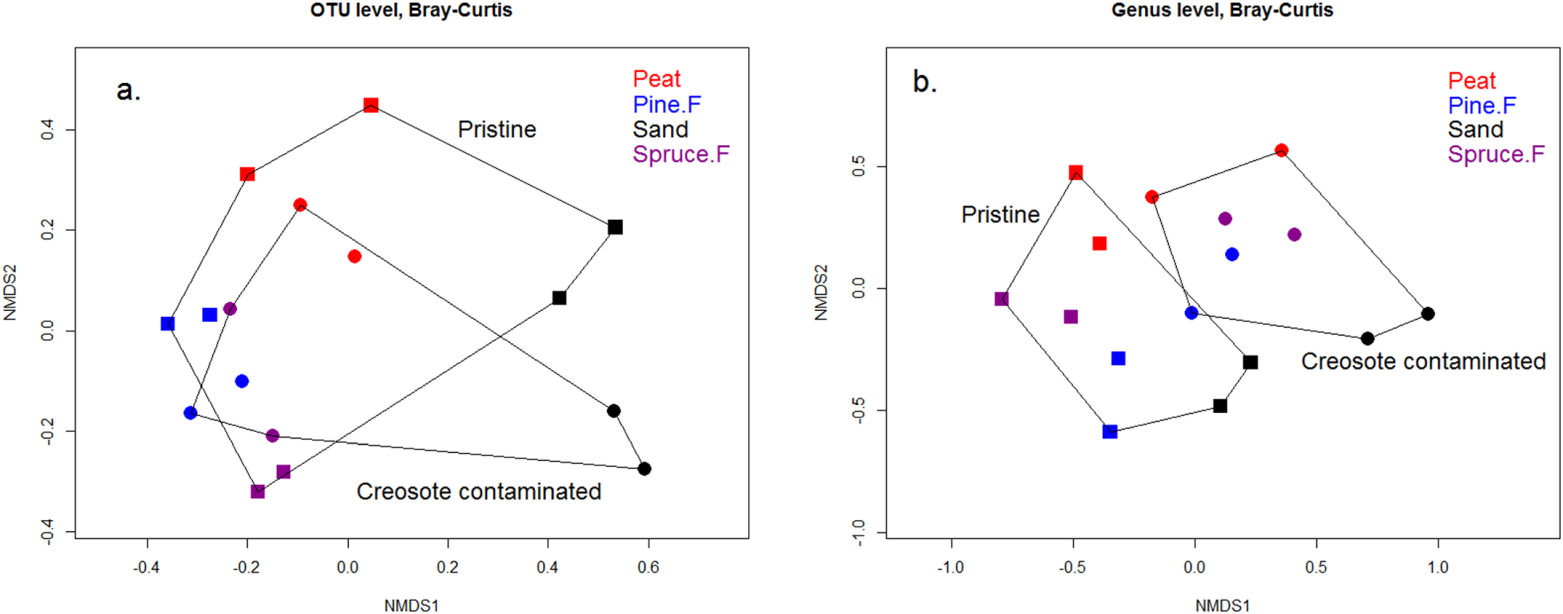
NMDS ordination (Bray-Curtis distance) of soil bacterial communities in the creosote contaminated and control soil samples. (a) The bacterial community composition in the two treatment groups overlap at the OTU level. (b) The communities are distinctively different at the genus level. For peat (Peat in the figure), pine forest soil (Pine.F) and spruce forest soil (Spruce.F), samples taken on week 31 (day91) were utilized, whereas week 27 (day 189) samples were used in the case of mineral soil (Sand).

The total bacterial community composition did not differ between creosote contaminated and pristine soils at the OTU level as revealed by the nonmetric multidimensional scaling (NMDS) ordination (Fig 1a) and permutational multivariate analysis of variance PERMANOVA (Table 3). The NMDS at the genus level, however, revealed distinctly different bacterial communities in the creosote contaminated and pristine soils (Fig 1b). PERMANOVA indicated that contamination and soil type had distinct main effects on microbial community at the genus level and without a strong interaction effect (Table 3). These findings underline that creosote contamination changed microbial community at the genus level regardless of the soil type.

### Community composition analysis of major bacterial phyla at OTU level

The major bacterial phyla detected from the soil samples were examined for their differences in community composition at the OTU level between the creosote contaminated and pristine soil samples. Since OTUs representing Bacteroidetes were not detected in two of the creosote contaminated samples, community composition analysis was not performed for Bacteroidetes. Consequently, the analyses were performed only for Proteobacteria and Actinobacteria.

#### Proteobacteria

**Fig 2:**
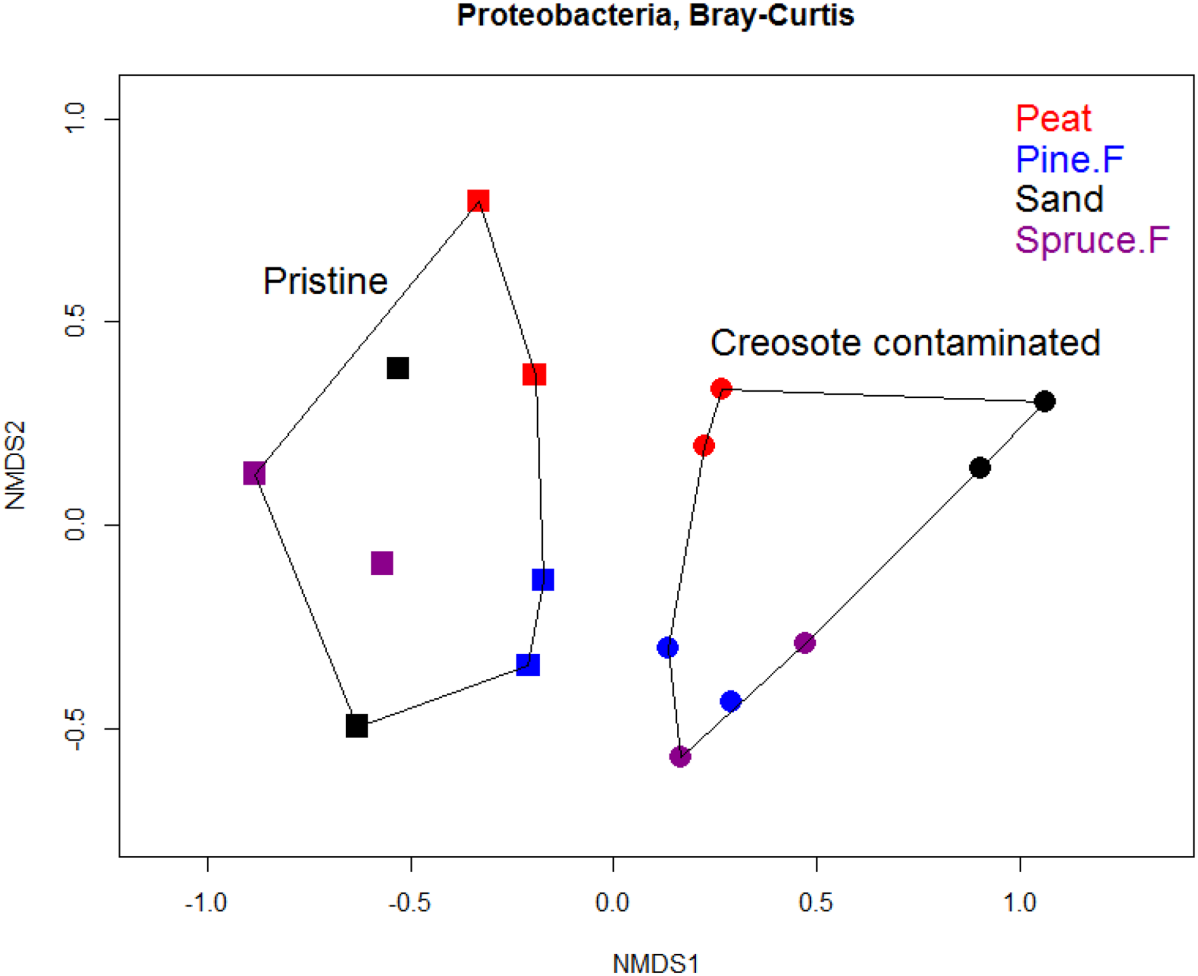
NMDS ordination of Proteobacterial OTUs in creosote contaminated and pristine (control) soil samples. The community composition is noticeably different in the two treatment groups. The ordination is based on Bray-Curtis dissimilarity metric.

Proteobacterial communities were distinctly different in contaminated than in control soils in NMDS (Fig 2). PERMANOVA revealed that the soil type and creosote contamination had strong individual effects on the Proteobacterial community composition, while the significance of the interaction term was ten times lower (Table 4).

**Table 4:** The effects of soil type and creosote contamination on Proteobacterial community composition at the OTU level in PERMANOVA.

	Df	Sum of squares	Mean square	F Model	R^2^	P value
Soil type (1)	3	1.50	0.50	2.13	0.28	0.001
Creosote addition (2)	1	0.82	0.82	3.50	0.15	0.001
1:2	3	1.06	0.35	1.51	0.20	0.010
Residuals	8	1.87	0.23		0.36	
Total	15	5.26			1.00	

#### Actinobacteria

**Fig 3:**
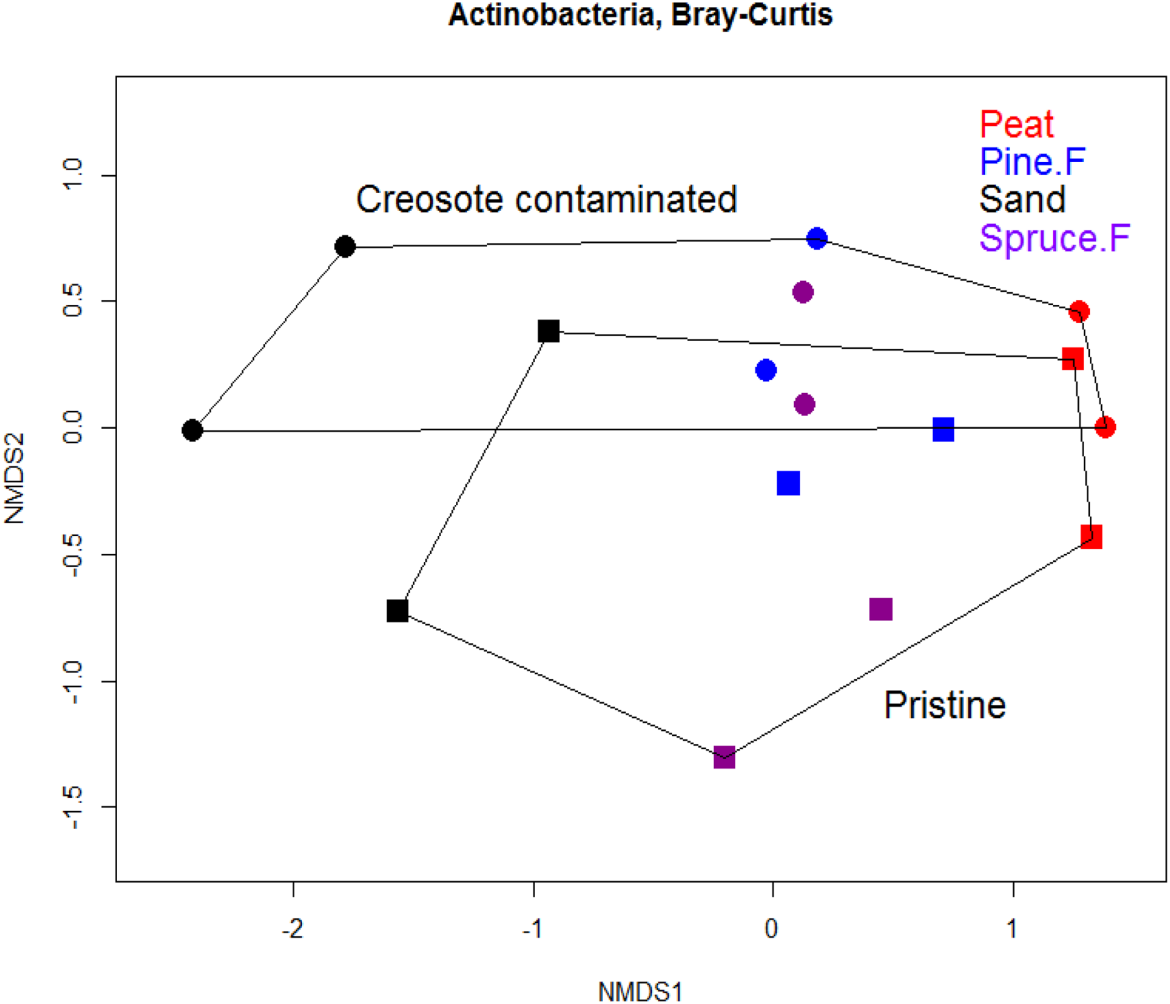
NMDS ordination of Actinobacterial OTUs in creosote contaminated and pristine soil samples. The community composition across the two treatment groups overlap partially. The ordination is based on Bray-Curtis dissimilarity metric.

Although the relative abundance of Actinobacteria decreased in creosote contaminated soil samples, it was observed that its community composition did not differ between the creosote contaminated and pristine soil samples (Fig 3). Instead, the soil type affected the Actinobacterial community composition found in that soil type (Table 5).

**Table 5:** The effects of soil type and creosote contamination on Actinobacterial community composition at the OTU level in PERMANOVA.

	Df	Sum of squares	Mean square	F Model	R^2^	P value
Soil type (1)	3	2.68	0.89	4.00	0.48	0.001
Creosote addition (2)	1	0.34	0.34	1.53	0.06	0.133
1:2	3	0.71	0.23	1.06	0.13	0.370
Residuals	8	1.78	0.22		0.32	
Total	15	5.51			1.00	

#### Temporal variation

**Fig 4:**
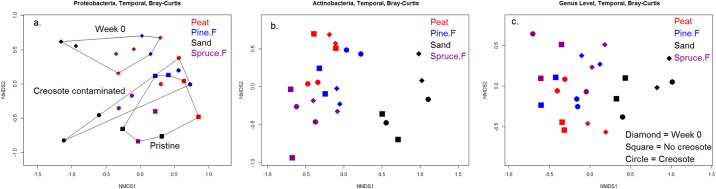
NMDS ordination revealing a temporal variation in the bacterial community structure. (a) Proteobacterial community on day 0 is distinctively different from those sampled at later time points (day 91 for peat, pine forest, and spruce forest soils, and day 189 for sand). Neither Actinobacterial community (b) nor the total bacterial community at genus level (c) varied with time.

The total bacterial, Proteobacterial and Actinobacterial communities sampled on day 0 were compared with those sampled later. Day 0 samples were compared with both creosote contaminated and pristine samples to determine the temporal community dynamics. Only Proteobacterial community on day 0 distinctly differed from the communities sampled at the later time points (Fig 4a). Neither total bacterial community nor Actinobacterial community changed during the experiment significantly (Fig 4b and 4c).

## Discussion

In the present study, we compared the responses of bacterial community to creosote contamination in four different types of soil. Our data indicates shifts in bacterial community composition and alterations in the relative abundances of three major bacterial phyla as a response to creosote contamination. The pollution-induced shift was attributable to an increase in the relative abundance of Proteobacteria, particularly Betaproteobacteria, and a decrease in the relative abundance of Actinobacteria and Bacteroidetes. These phyla are the major bacterial phyla present in soil as well as on human skin and other biological samples [49–51]. Because they have been recently linked to human immune function and chronic diseases [25–27, 52], a major change in their community composition in the environment can potentially affect human exposure to them and, eventually, lead to modulated human immune system functions [27, 31]. As far as we know, this idea that we call the altered environmental microbiome hypothesis has never been proposed nor tested. The first step to evaluate the hypothesis is therefore to perform a literature review on the potential connections between health-associated and pollution-induced changes.

While several studies have investigated and pointed out the alteration of the entire bacterial community as a result of PAH pollution, only a handful of studies have focused on pollution-induced changes in Proteobacterial, Bacteroidetes, and Actinobacterial communities in surface soil [1, 12, 53–58]. Those studies have revealed that Proteobacteria were more abundant in PAH polluted soils compared to pristine soils. Phylum Bacteroidetes, in contrast, either decreased in abundance [55] or was not studied. The relative abundance of Actinobacteria was usually lower in contaminated soil than in non-contaminated soil [55], but its abundance is also dependent on the PAHs involved[57] (Table 6). The increase in the abundance of Proteobacteria and the decrease in Actinobacteria and Bacteroidetes are consistent with our findings. As the studies listed in Table 6 cover a wide range of geographic areas and land use histories, it seems plausible to assume that shifts in the abundance of Proteobacteria, Actinobacteria, and Bacteroidetes are a typical consequence of PAH pollution in soil.

**Table 6:** Alteration in the abundance of Proteobacteria, Actinobacteria, and Bacteroidetes in PAH polluted soil as reported in earlier studies.

Sample type and location	Proteobacteria	Bacteroidetes	Actinobacteria	Remarks, subphyla or other altered phyla
Wood impregnation site, Finland [38]	+	NS	-	-TM7, Planctomycetes
Soil artificially contaminated with pyrene during composting, China [39]	+	NS	+	
contaminated soil from former manufactured-gas plant site, USA [12]	+	-(Not significant)	NS	+ Sphingomonas
Soil from constructed wetland from road run-off spiked with PAH, France [40]	+	NS	NS	+ Betaproteobacteria
Soil from timber preservation facility spiked with PAHs, Ireland [41]	+	-	+ or -	Actinobacteria decreased in phenanthrene contaminated and increased in fluoranthene contaminated soils + Gammaproteobacteria
Industrial creosote contaminated soil, Portugal [42]	+	-	-	+ Beta and Gammaproteobacteria, decrease in Bacteroidetes and Actinobacteria attributed to added non-ionic surfactant
Effect of sunflower rhizosphere in creosote polluted clay and agricultural soil, Spain [43]	+	-	NC	
Windrow treatment of soil contaminated by 2,3,4-ring PAHs, France [1]	+	NS	-	+ Gamma, Betaproteobacteria appeared at the end of treatment

NS represents not studied, + implies increased abundance,–denotes decreased abundance, and NC denotes no change.

The most common method to compare soil remediation in different treatments is to take samples at selected time intervals and compare the measured variables, such as microbial communities, between sampling dates [38, 59–61]. As the rate of soil remediation is dependent on soil type, it is conceivable that only the time-dependent changes are reported or noticed, and the microbial community changes connected to actual degradation are ignored. Since chemical analyses were done at certain intervals and the results were then compared to 20% degradation level, the abovementioned concern was overcome in this study. Therefore, changes in the relative abundance of Bacteroidetes, Actinobacteria, Proteobacteria, and particularly Betaproteobacteria were detected.

The observed higher relative abundance of the subphylum Betaproteobacteria as a result of 20% PAHs degradation may turn out to be an important observation for several reasons. Earlier findings suggest that Betaproteobacteria are indicators of the endpoint of fast bioremediation in soil [1]; in this study, soil remediation ceased before the last sampling in organic soils (data not shown). Interestingly, an increase in Betaproteobacterial community has been associated with degradation of PAHs [1] [55]. Betaproteobacteria are also enriched in the faeces of diabetic people compared to non-diabetic individuals [62] meaning that Betaproteobacteria have been associated with both soil pollution and health. Based on the current and earlier findings [1], it is worthwhile to study how common and permanent changes in the abundance of Betaproteobacteria are in contaminated environments. If the changes persist, health-perspective should be further investigated, e.g., whether the same species are enriched in polluted soil and diabetics.[55] At the level of the whole phylum Proteobacteria, a similar but less sharp increase was reminiscent of the increase in the abundance of Proteobacteria in the household dust and biological samples that have been associated with several diseases [62–67]. Hence, it should be considered whether pollution-induced changes in Proteobacterial community composition in areas prone to anthropogenic PAHs could be linked to chronic diseases [62–67].

In parallel with Proteobacteria, Bacteroidetes is another dominant phylum in the environment as well as in the human gut [68–70]. Several members of Bacteroidetes have been reported to be sensitive to hydrocarbons and are thought to be outcompeted by other hydrocarbon-degrading bacteria leading to their reduced abundance [71], which could have led to their decreased relative abundance in creosote contaminated soils in this study. Interestingly, in human body, Bacteroidetes are involved in the maintenance of immune system by promoting various regulatory components [72, 73], and therefore their reduced abundance has been implicated to have a role in immune disorders and other inflammatory diseases [26, 67, 74–76].

We also observed that there was a relative decrease in Actinobacterial abundance in creosote contaminated compared to pristine soils. This finding is in contrast with studies that pointed out that Actinobacteria contains efficient degraders of PAH and their abundance is increased in PAH polluted soils [54, 77, 78]. However, two other studies have reported a decreased abundance of Actinobacteria in creosote contaminated soil [53, 79], which is in accordance with findings of this study. A plausible explanation for the varying results is that the PAH-degrading taxa within Actinobacteria were not ubiquitous in this study. Nevertheless, the diminished abundance of Actinobacteria has been observed to have clinical significance in non-communicable diseases [62, 80]. Therefore, a pollution-induced reduction in the supply of Bacteroidetes and Actinobacteria from the environment may be a part of a novel mechanism how environmental PAH pollution is associated with health, and more specifically, with immune disorders. As PAH compounds are a major ingredient of urban smog, the adverse effects of altered soil microbial communities may be common in densely populated areas.

Recently, it has become increasingly more evident that environmental biodiversity contributes in shaping the human microbiota, and that differences in bacterial abundance and diversity are associated with health outcomes [27, 81–86]. Consequently, shifts in environmental microbial community in polluted areas may lead to similar effects in human microbial community and subsequent health consequences. We are well aware of the fact that not all members belonging to the phyla we studied have associations with human health and therefore the shifts in abundance and alteration in the community composition have to be established at a finer taxonomic level to draw definitive conclusions about the association between pollution and shifts in health associated environmental microbiome. In this study, however, we did not search for shifts in abundance and community composition beyond the phylum level (except for Proteobacteria) to avoid erroneous conclusions drawn from the shallow sequencing depth of 454-pyrosequencing technology. Therefore, future studies must utilize more powerful next generation sequencing methods to explore the relationship between pollution induced shifts in the alteration of bacterial communities and human health.

We hypothesized that PAH pollution will cause shift in the abundance and community composition of environmental bacterial phyla that have known associations with human health. Our findings that the relative abundance of Proteobacteria is increased and that of Actinobacteria and Bacteroidetes is decreased after PAH exposure supports our altered environmental microbiome hypothesis. The next step is to study the differences in skin and gut microbiome of people living in severely PAH-exposed versus mildly PAH-polluted areas. If differences in Proteobacterial, Actinobacterial, and Bacteroidetes community on human skin and in stool samples are associated with PAHs or other environmental pollutants, this would further strengthen our altered microbiome hypothesis, and the changes could well turn out to be important factors in explaining the increase in the frequency of immune mediated and other non-communicable diseases among city dwellers.

In parallel to studying the direct and indirect impacts of PAH exposure, it is crucial to know whether shifts in health-associated microbial community are restricted to PAHs and if the changes are irreversible. As this has not been the focus of previous research, a comprehensive answer requires novel research. However, one of our previous studies made with diesel oil indicates that changes in Proteobacterial community are persistent after soil remediation; Gammaproteobacteria were less frequent in naturally remediated diesel contaminated soil as compared to similar pristine soil samples [38]. As the relative abundance of Proteobacteria in general and Gammaproteobacteria in particular has been associated with the quality of living environment in healthy but not atopic individuals [27, 52], the finding of our previous study [38] is in accordance with our novel altered environmental microbiome hypothesis.

Our findings indicate that PAH pollution alters the community composition and changes the relative abundance of soil Proteobacterial, Actinobacterial, and Bacteroidetes communities. As environment can tune bacterial community on human skin [27], alterations in environmental microbiome can be an important component explaining why PAH pollution is connected to several health deficits. It may be worthwhile to consider if other pollutants change health-associated environmental microbiome similarly and also if exposure to PAHs alters human microbial community on skin and other tissues. For these reasons, the altered environmental microbiome hypothesis proposed in this study can supplement the currently prevailing direct toxin effect hypothesis and encourage further studies to explore a potential new dimension in interrelations between environmental pollution, microbiota, and human health.

## Supporting information

S1 Table Sum of total PAHs concentration (μg/kg) before and after natural attenuation. PAHs were measured after 28, 91 and 189 days.
(DOCX)
