# Supplementary material for: The abundance of health-associated bacteria is altered in PAH polluted soils—Implications for health in urban areas?
Source: PLoS One. 2017 Nov 16;12(11):e0187852. doi: 10.1371/journal.pone.0187852 (PMC5690629; doi:10.1371/journal.pone.0187852)
Supplement: S1 Table — PAHs were measured after 28, 91 and 189 days. (DOCX) [file pone.0187852.s001.docx]

|  |  | |  |  |
| --- | --- | --- | --- | --- |
| Soil type | **Beginning (day 28)** | | **Day 91** | **Day 189** |
| Sand 1 | 268465 | | 325119 | 133795.9 |
| Sand 2 | | 297235.6 | 282268.8 | 156574.8 |
| Spruce forest soil 1 | | 1729647 | 305883.1 | 76749.06 |
| Spruce forest soil 2 | 1851362 | | 1195448 | 934095.6 |
| Pine forest soil 1 | 523038.28 | | 326153.5 | 172839.1 |
| Pine forest soil 2 | 1730658.7 | | 846964.3 | 346904.5 |
| Peat 1 | 2949917.7 | | 1134664 | 465802 |
| Peat 2 | 3112694.4 | | 1602742 | 544774.4 |
